# Gender differences in quality of life in coronary artery disease patients with comorbidities undergoing coronary revascularization

**DOI:** 10.1371/journal.pone.0234543

**Published:** 2020-06-17

**Authors:** Tom H. Oreel, Pythia T. Nieuwkerk, Iris D. Hartog, Justine E. Netjes, Alexander B. A. Vonk, Jorrit Lemkes, Hanneke W. M. van Laarhoven, Michael Scherer-Rath, Mirjam A. G. Sprangers, José P. S. Henriques

**Affiliations:** 1 Department of Medical Psychology, Amsterdam University Medical Centers, Location Academic Medical Center, University of Amsterdam, Amsterdam Public Health Research Institute, Amsterdam, The Netherlands; 2 Faculty of Philosophy, Theology and Religious Studies, Radboud University Nijmegen, Nijmegen, The Netherlands; 3 Department of Cardio-thoracic Surgery, Amsterdam University Medical Centers, Location Vrije Universiteit Amsterdam, Amsterdam, The Netherlands; 4 Department of Cardiology, Amsterdam University Medical Centers, Location Vrije Universiteit Amsterdam, Amsterdam, The Netherlands; 5 Department of Medical Oncology, Cancer Center Amsterdam, Amsterdam University Medical Centers, University of Amsterdam, Amsterdam, The Netherlands; 6 Department of Cardiology, Amsterdam University Medical Centers, Location Academic Medical Center, University of Amsterdam, Amsterdam, The Netherlands; Qazvin University of Medical Sciences, ISLAMIC REPUBLIC OF IRAN

## Abstract

In comparison to male patients with coronary artery disease, female patients suffer from more comorbidities, experience symptoms of coronary artery disease differently and report poorer health-related quality of life (HRQoL) after coronary revascularization. However, there is limited data on the impact of comorbidity burden on the recovery in HRQoL in female and male patients. We investigated the impact of comorbidity burden on the change in HRQoL following coronary revascularization in female patients versus male patients. 230 patients (60 female) with coronary artery disease were assessed before, and two weeks, three months and six months after coronary revascularization. Disease-specific HRQoL was measured with the Short-Form Seattle Angina Questionnaire. Physical and mental health was measured with the Short-Form Health Survey. Comorbidity burden was assessed by the total number of identified comorbidity conditions and by the Charlson comorbidity score. Linear mixed models were used to estimate the effects of time, gender and comorbidity burden on HRQoL. Whereas HRQoL improved after coronary revascularization in all patients, female patients reported poorer physical health and disease-specific HRQoL and their physical health improved more slowly than male patients. A higher comorbidity burden was related with poorer physical health and disease-specific HRQoL in male patients, but not in female patients. A higher comorbidity burden was associated with slower improvement in HRQoL for both female and male patients. Female patients reported poorer HRQoL and their physical health improved more slowly after coronary revascularization, irrespective of comorbidity burden. Higher comorbidity burden was associated with poorer physical health and disease-specific HRQoL in male patients only. Our results indicate that female and male patients recover differently after coronary revascularization. These findings highlight the importance of comorbidity- and gender-specific approaches for evaluating coronary artery disease and coronary revascularization procedures.

## Introduction

Coronary artery disease (CAD) is the leading cause of death and disability in the Western world [1]. The treatment of CAD aims at symptom relief and improvement of health-related quality of life (HRQoL) [2, 3]. Measures of HRQoL have been found to be valid indicators of the burden of disease and the effectiveness of treatments [4, 5].

As CAD patients are becoming older, they are more likely to suffer from comorbidities which increases the overall disease burden and may negatively impact HRQoL [6–9]. Female CAD patients are generally older compared to their male counterparts, tend to have more comorbidities, and report a higher comorbidity burden [10–13]. Furthermore, female patients experience symptoms of CAD differently than male patients [11, 14]. These gender differences in the prevalence of comorbidities and CAD symptoms may (in part) explain why female patients report poorer HRQoL than male patients [15–17]. Many factors may play a role in selecting patients for coronary revascularization and patients of either gender have mainly been regarded the same. However, recent insights allow for a more individualized decision-making, especially as the main goal of planned revascularization is to reduce symptoms, allowing recovery to resume former normal activities. Since female and male patients may experience symptoms differently, a gender specific approach in coronary revascularization may be of great clinical importance. There is however limited data about gender differences on the impact of comorbidity burden on HRQoL after coronary revascularization procedures.

This study aims to investigate the impact of comorbidity burden on changes in HRQoL following coronary revascularization in female versus male CAD patients.

## Materials and methods

### Patients

All patients were recruited at the cardiology departments of the Amsterdam University Medical Centers: Academic Medical Center (AMC) and VU Medical Center (VUmc) locations. All patients were planned for coronary revascularization procedure after being discussed in the multidisciplinary “heart teams”. Patients were eligible if they were 18 years or older, had stable CAD and were scheduled for elective coronary artery bypass graft (CABG) or elective percutaneous coronary intervention (PCI). Patients had to have at least one comorbidity (see S1 Table in supporting information for the included comorbidity conditions). Patients were excluded if they were unable to complete questionnaires due to language or cognitive problems.

### Procedure

Patients completed questionnaires on HRQoL one week before, and two weeks, three months and six months after their coronary revascularization. Demographic information was collected at baseline. Patients completed the questionnaires on paper or online. Written informed consent was obtained from all patients. The study was approved by the ethics committee at the Amsterdam University Medical Centers. As the central ethics committee decided that the Medical Research Involving Human Subjects Act did not apply, the study was exempted from further ethical assessment.

### Assessment of HRQoL

#### Generic HRQoL

Generic HRQoL was assessed using the Short-Form Health Survey [18]. The questionnaire consists of 36 items that form eight subscales, which can be integrated into a *physical health* (PCS) and *mental health* (MCS) component score. The component scores are transformed into a 0–100 scale, with higher scores indicating better (physical or mental) health.

#### Disease-specific HRQoL

Disease-specific HRQoL was assessed using the Short-Form Seattle Angina Questionnaire (SAQ) [19]. The SAQ assesses patients’ physical limitations caused by CAD, the frequency of and recent changes in their angina symptoms, their satisfaction with treatment, and the degree to which they perceive their disease to affect their quality of life. An overall summary SAQ score was derived by taking the average of the 3 domain scores. SAQ scores ranged from 0 to 100, where higher scores indicate better disease-specific HRQoL (e.g., less physical limitations, less angina symptoms, and better quality of life).

### Assessment of comorbidity burden

Patients’ comorbidity conditions were identified using the hospitals electronic medical records. If records were unclear, they were checked by two medical specialists. Comorbidity burden was operationalized using two methods:

#### Total comorbidity conditions

For each patient we summed all identified comorbidity conditions and categorized them into: *low* (summed comorbidities = one), *middle* (summed comorbidities = two), and *high* (summed comorbidities = three or higher). The higher the category, the higher the comorbidity burden.

#### Charlson comorbidity category

The Charlson comorbidity category is based on the Charlson comorbidity score [20], which quantifies the mortality risk of patients who have a range of comorbidity conditions. Each patient’s identified comorbidity conditions were first classified into 17 comorbidity conditions using the diagnostic codes of the 10th revision of the International Statistical Classification of Diseases and Related Health Problems (ICD-10 [21]). Each classified comorbidity condition was assigned a weight from one to six, with a weight of six representing the highest mortality risk. Next, weighted comorbidity conditions were summed and categorized into: *low* (sum weighted comorbidities = zero), *middle* (sum weighted comorbidities = one), and *high* (sum weighted comorbidities = two or higher). The higher the category, the higher the comorbidity burden.

### Confounding variables

*Age* (in years; self-reported at baseline) and *intervention type* (PCI/CABG; identified using the hospitals electronic medical records) were included as possible confounders as they are associated with comorbidity burden and with HRQoL [6, 7, 22].

### Statistical analysis

We used linear mixed models to investigate if HRQoL changed over time for all patients, if there were different patterns of change in HRQoL between male and female patients, if the impact of comorbidity burden on HRQoL differed between male and female patients, and if there was a different pattern of change over time in HRQoL among patients with different levels of comorbidity burden. All analyses were adjusted for age and intervention type. Linear mixed models allow for missing HRQoL measurements and provide valid estimates without the need for imputation of data. For both the total comorbidity conditions and the Charlson comorbidity category we estimated a separate model, resulting in two estimates for each HRQoL measure. Patient’s intercepts were modelled as random effects. All linear mixed models were estimated using the R-package *lme4* version 1.1–16 [23].

## Results

### Patients

**Fig 1:**
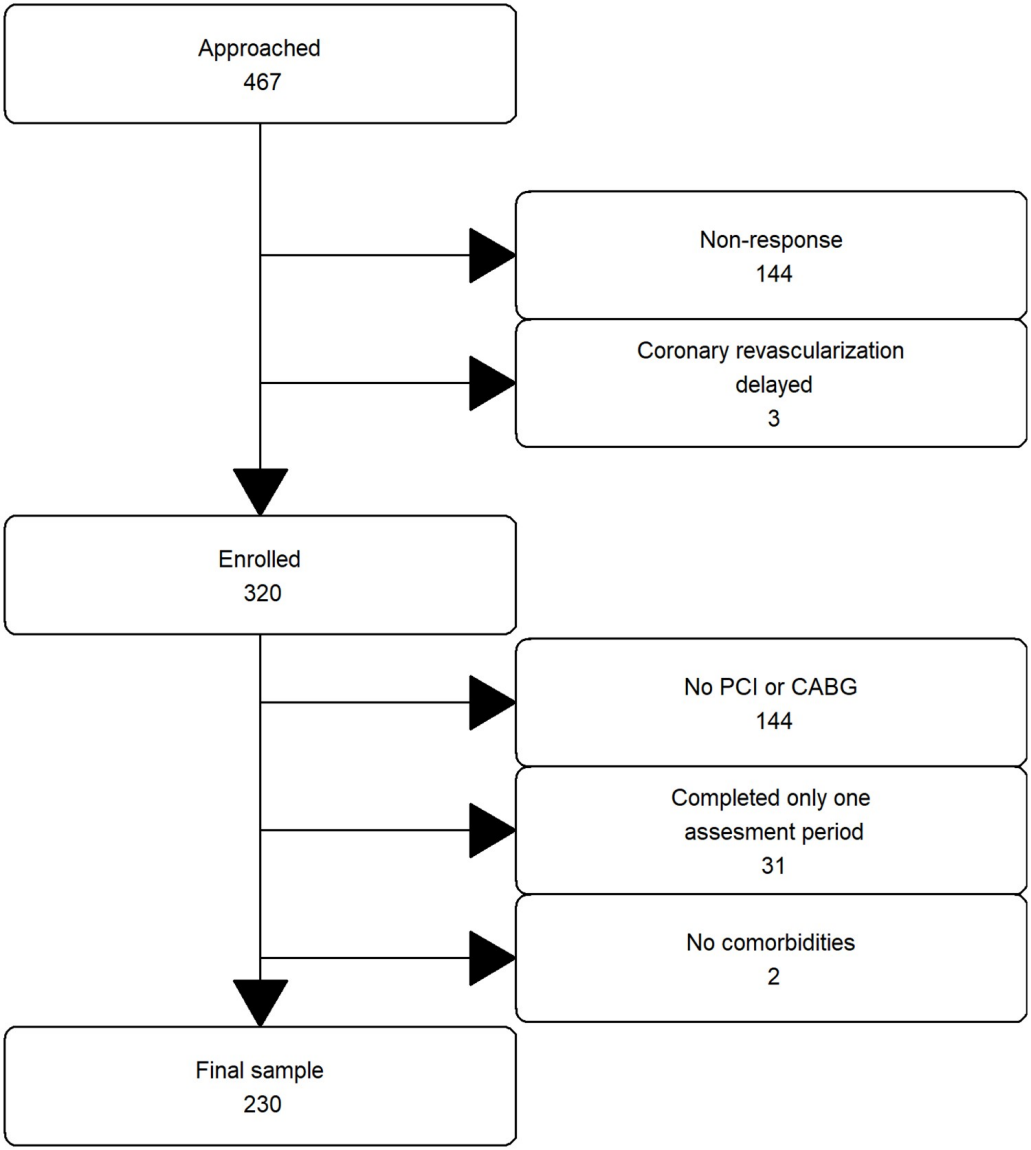
Patient inclusion and exclusion. PCI indicates percutaneous coronary intervention. CABG indicates coronary artery bypass graft.

Data collection took place from September 2015 until March 2018. A total of 467 patients were approached for the study, of whom 144 patients did not respond (31%) and three patients were excluded because their coronary revascularization was delayed. From the remaining 320 patients, 57 patients were excluded because they only underwent a diagnostic procedure, 31 patients were excluded because they responded to questionnaires at only one assessment period, and two patients were excluded because no comorbidities were identified. This resulted in a final sample size of 230 (see Fig 1 for the inclusion flow chart). There were no differences in gender and age between the 90 excluded patients and the final sample. Of the 230 patients, 9 missed the baseline, 9 missed the second, 34 missed the third and 35 missed the fourth assessment. Patients’ background characteristics can be found in Table 1 and the specific comorbidity conditions in S1 Table in the supporting information. The mean age was 68 (SD = 9.37), and patients had two comorbidities on average (SD = 1.16). There were no gender differences in age, number of comorbidities and comorbidity burden, however male patients were more likely to have diabetes than female patients.

**Table 1:** Patient characteristics (N = 230).

Characteristics	Female patients (%)	Male patient (%)	*χ*^2^ (*p* value)
Total, n (%)	59 (26)	171 (74)	
Mean age (SD)	68 (9.98)	68 (9.18)	43.13 (0.42)
Intervention type:			
PCI	53 (90)	129 (75)	4.67 (0.03*)
CABG	6 (10)	42 (25)	
Mean number of comorbidities (SD)	2 (1.04)	2 (1.20)	.29 (0.77)
Comorbidity conditions:			
Diabetes mellitus	20 (34)	86 (50)	4.11 (.04*)
BMI > 30 kg/m^2^	19 (32)	62 (36)	0.16 (.68)
Joint disease	20 (34)	37 (22)	2.91 (.09)
Pulmonary disease	14 (24)	37 (22)	.02 (.88)
Other chronic diseases	22 (37)	73 (43)	.33 (.57)
Total comorbidity conditions:			
1	22 (25)	66 (75)	.70 (.71)
2	19 (32)	62 (36)	
>2	18 (31)	43 (25)	
Charlson comorbidity categories:			
0	23 (39)	51 (30)	1.83 (.40)
1	27 (46)	86 (50)	
>1	9 (15)	34 (20)	

*P values < 0.05 were considered significant.

*PCI* indicates elective percutaneous coronary intervention. *CABG* indicates elective coronary artery bypass graft. *BMI* indicates Body mass index, calculated as weight in kilograms divided by height in meters squared, rounded to 1 decimal. *Joint disease* indicates rheumatism, arthritis, osteoarthritis, and gout. *Pulmonary disease* indicates asthma, chronic obstructive pulmonary disease, and bronchiectasis. *Other chronic diseases* are indicated in S1 Table in the supporting information. A higher score on *total comorbidity conditions* and *Charlson comorbidity categories* indicates a higher comorbidity burden.

### The impact of comorbidity burden on HRQoL between female and male patients

#### Impact on physical health

**Fig 2:**
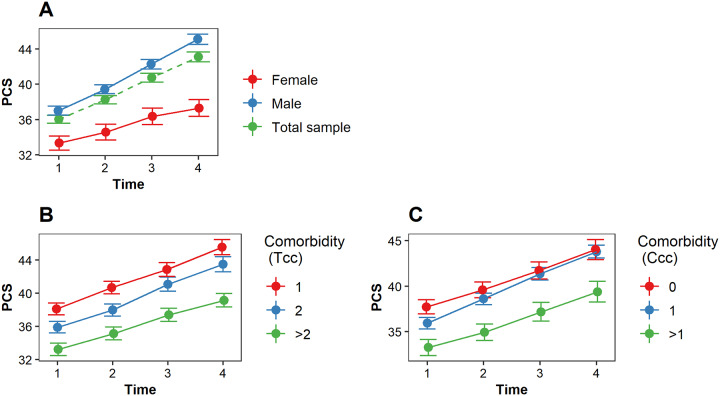
The estimated PCS (physical health) score over time. (A) The estimated PCS score over time for *female patients* (red), *male patients* (blue) and *the total sample* (green). (B) The impact of *comorbidity burden* (total comorbidity conditions) on PCS over time; (C) The impact *of comorbidity burden* (Charlson comorbidity categories) on PCS over time. *Tcc* indicates the total comorbidity conditions; *Ccc* indicates the Charlson comorbidity categories. A higher Tcc and Ccc indicates a higher comorbidity burden.

Physical health improved for all patients, however female patients reported poorer physical health than male patients and reported slower recovery (change over time) in physical health (Fig 2A, Table 2). A higher comorbidity burden was associated with poorer physical health in male patients but not in female patients according to the total comorbidity conditions (Table 2). This relationship was not found for the Charlson comorbidity category (Table 2). There was no impact of comorbidity burden on the recovery in physical health (total comorbidity burden and Charlson comorbidity burden, Fig 2B and 2C, Table 2). The potential confounders age and intervention type were not significantly associated with physical health.

**Table 2:** Parameter estimates of the linear mixed models for each HRQoL measure.

Variables	β	SE	*P* value
***Physical Health (PCS)***			
Time	2.56	4.01	< .001*
Gender (female)	-5.13	1.77	< .001*
Time × gender (female)	-1.16	0.53	.030*
Comorbidity burden:			
Total comorbidity conditions	-1.60	0.28	< .001*
Charlson comorbidity categories	-4.64	1.84	.001*
Comorbidity burden × gender(female):			
Total comorbidity conditions	5.94	3.04	.008*
Charlson comorbidity categories	5.64	3.76	.156
Comorbidity burden × time:			
Total comorbidity conditions	-0.20	0.47	.840
Charlson comorbidity categories	-0.11	0.56	.610
***Mental health (MCS)***			
Time	1.64	0.29	< .001*
Gender (female)	-1.07	1.97	.245
Time × gender (female)	-0.18	0.56	.650
Comorbidity burden:			
Total comorbidity conditions	-1.06	1.77	.343
Charlson comorbidity categories	-2.57	2.02	.091
Comorbidity burden × gender(female):			
Total comorbidity conditions	-0.35	3.39	.250
Charlson comorbidity categories	-1.59	4.12	.077
Comorbidity burden × time:			
Total comorbidity conditions	-0.64	0.51	.221
Charlson comorbidity categories	0.13	0.59	.310
***Disease-specific HRQoL (SAQ)***			
Time	7.57	0.54	< .001*
Gender (female)	-4.86	3.41	< .001*
Time × gender(female)	-1.34	1.04	.211
Comorbidity burden:			
Total comorbidity conditions	-6.94	3.13	.362
Charlson comorbidity categories	-9.64	3.58	.029*
Comorbidity burden × gender(female):			
Total comorbidity conditions	18.28	5.89	.001*
Charlson comorbidity categories	17.26	7.35	.209
Comorbidity burden × time			
Total comorbidity conditions	0.15	0.94	.032*
Charlson comorbidity categories	0.93	1.10	.636

*P values < 0.05 were considered significant.

#### Impact on mental health

**Fig 3:**
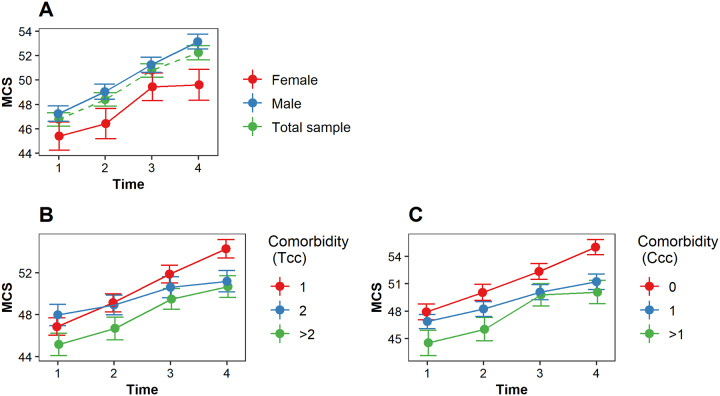
The estimated MCS (mental health) score over time. A higher MCS score indicates a better mental health. (A) The estimated MCS score over time for female patients (red), male patients (blue) and the total sample (green). (B) The impact of comorbidity burden (total comorbidity conditions) on MCS over time; (C) The impact of comorbidity burden (Charlson comorbidity categories) on MCS over time. *Tcc* indicates the total comorbidity conditions; *Ccc* indicates the Charlson comorbidity categories. A higher Tcc and Ccc indicates a higher comorbidity burden.

Mental health improved significantly over time for all patients. There was no difference in the pattern of change over time between male and female patients (Fig 3A, Table 2). Patients with a low, middle or high comorbidity burden did not differ in mental health. Also, we did not find an impact of comorbidity burden on the change in mental health (total comorbidity burden and Charlson comorbidity burden, Fig 3B and 3C, Table 2). From the potential confounders (i.e., age and intervention type) only age was significantly associated with mental health, with older patients reporting better mental health than younger patients.

#### Impact on disease-specific HRQoL

**Fig 4:**
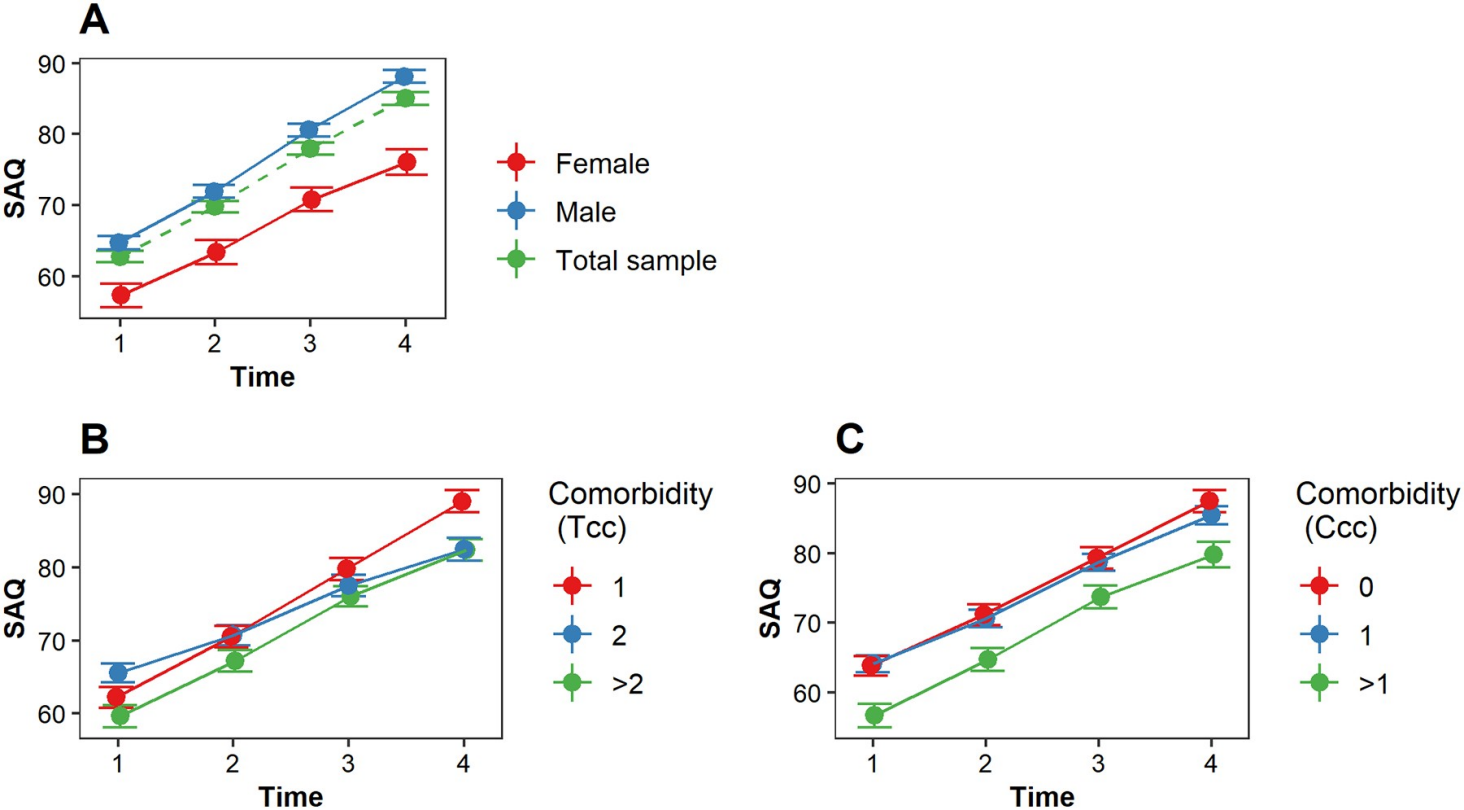
The estimated SAQ (disease-specific HRQoL) score over time. A higher SAQ score indicates a better disease-specific HRQoL (e.g., less physical limitations, less angina symptoms, and better quality of life). (A) The estimated SAQ (disease-specific HRQoL) score over time for female patients (red), male patients (blue) and the total sample (green). (B) The impact of comorbidity burden (total comorbidity conditions) on SAQ over time; (C) The impact of comorbidity burden (Charlson comorbidity categories) on SAQ over time. *Tcc* indicates the total comorbidity conditions; *Ccc* indicates the Charlson comorbidity categories. A higher Tcc and Ccc indicates a higher comorbidity burden.

Disease-specific HRQoL improved significantly over time for all patients. Female patients reported significantly poorer disease-specific HRQoL than male patients. However, the pattern of change in disease-specific HRQoL did not differ between female and male patients (Fig 4A, Table 2). A higher comorbidity burden was associated with poorer disease-specific HRQoL in male patients but not in female patients (total comorbidity conditions, Table 2). Such relationship was not found with the Charlson comorbidity category (Table 2). Patients with a higher comorbidity burden reported slower improvement in disease-specific HRQoL than patients with a lower comorbidity burden (total comorbidity conditions, Fig 4B, Table 2). This relationship was not found for the Charlson comorbidity category (Fig 4C, Table 2). The potential confounders age and intervention type were not significantly associated with disease specific HRQoL.

## Discussion

The present study investigated the impact of comorbidity burden on the change in HRQoL in female versus male patients after coronary revascularization. The HRQoL of all patients improved after coronary revascularization. However, female patients reported poorer physical health and disease-specific HRQoL than male patients and their physical health improved more slowly after revascularization than that of male patients. Surprisingly, we found that a higher comorbidity burden was associated with poorer physical health and poorer disease-specific HRQoL in male patients but not in female patients. Lastly, a higher comorbidity burden was associated with slower improvement in disease-specific HRQoL following coronary revascularization for both female patients and male patients.

While female patients reported worse physical health and disease-specific HRQoL than male patients, female patients did not differ from male patients in mental health. These results are in contrast to previous findings in both general and CAD populations where female reported poorer mental health than male [15, 24].

We also found that the physical health of female patients recovered more slowly than that of male patients following coronary revascularization. Our findings are in line with those of Sajobi and colleagues [25] who investigated long term changes in HRQoL, i.e., one to five years after PCI, CABG or medical management of CAD. Patients with persistently poorer HRQoL over a long period of time were more often female.

In contrast to our findings, a previous study reported a negative impact of comorbidity on the HRQoL in both male and female patients. In this previous study [6] they measured the HRQoL of 408 Norwegian patients two and a half years after MI and investigated the association of physical (PCS) and mental health scores (MCS) with demographic variables, comorbid conditions (COPD, diabetes, previous MI, hypertension) and other clinical variables. Similar to our study, researchers found that females scored lower than males on PCS and that comorbidities (i.e., COPD) had a negative impact on the PCS scores of male patients. Furthermore, researchers also found no gender differences on MCS scores. However, in contrast to our study, comorbidities (i.e., COPD, stroke, previous MI) also had a negative impact on the PCS scores of female patients. This discrepancy with our findings may be explained by the differences in heart condition (MI vs stable CAD), treatment procedure (emergency vs planned hospitalization), included comorbidity conditions, and study design (cross-sectional vs longitudinal measurements).

Also of interest, we found that a higher comorbidity burden was accompanied with a slower improvement (change over time) in disease-specific HRQoL, whereas the rate of improvement in physical health was not related to comorbidity burden. It seems that comorbid conditions not only have worsened CAD symptoms but also have impeded recovery of disease-specific HRQoL. Interestingly, the most frequent comorbid condition in our sample was diabetes, which has been shown to be associated with lower recovery in HRQoL than in patients without diabetes [26].

### Implications for research and practice

Our results indicate that HRQoL following coronary revascularization is experienced differently by female than by male patients. We also found a difference in comorbidity burden between female and male patients. Specifically, comorbidity burden was found to be more strongly associated with worse HRQoL in male patients than in female patients. Although we cannot entirely explain this finding, depression may be involved as an intermediary variable between gender and comorbidity burden [25]. Since research into gender differences and disease burden is in its infancy, more research is needed to confirm our findings and explore the causes of gender differences in the impact of comorbidity burden on HRQoL after coronary revascularizations.

We encourage health care providers to be alert to comorbidity burden among their CAD patients who are scheduled to undergo coronary revascularization, as these patients might require extra care. Our results also emphasize the importance of gender-specific health care approaches before and after coronary revascularization. Future studies could explore whether comorbidity- and gender-specific health care approaches following coronary revascularization would increase the effectiveness of coronary revascularization. Such research is needed to ensure that CAD patients receive appropriate and patient-tailored care.

### Limitations and strengths

This study has several limitations. We only included patients with at least one comorbidity and therefore could not contrast these patients with those without comorbidity. Further, the number of female patients was small, and accounted for only 26% of the sample, although this percentage is in line with a common CAD population. Post-hoc power analysis revealed that, with a sample size of 60 and a two-sided alpha of 0.05 we were able to detect a standardized mean difference of 0,52 between male and female patients with 80% power [27, 28]. The sample of 59 female patients can therefore be considered as sufficiently large. Moreover we found that significantly fewer female patients were affected by diabetes than male patients (34% and 50% respectively.). This difference reflects the differential prevalence rates of diabetes in the Dutch population[29], thereby rendering selection bias unlikely. We did not investigate the socio-economic status (SES) of our patients. SES may play an important role in the course and prevalence of CAD and the effect of SES on CAD may differ between male and female patients [30]. In the present study we only collected data about the level of education, which is an indicator of SES. However we did not find significant differences between male and female patients in educational level (data not shown). The Charlson comorbidity score was originally designed to predict mortality risk, not to assess the impact of comorbidities on HRQoL in general, nor that of CAD patients, in particular [20]. The weight assigned to the comorbidity conditions may therefore not reflect their impact on HRQoL. Furthermore, although widely used, the Charlson comorbidity index does not include all comorbidity conditions. We therefore added the total number of comorbidity conditions in an attempt to include all comorbidities and to provide an alternative comorbidity burden score. However, it should be noted that gender specific comorbidities; e.g., those due to post-menopausal status in female patients [31], were not included. Finally, this study was not primarily designed to test the differential effects of comorbidity burden on the change in HRQoL of male and female CAD patients undergoing revascularization, but rather to improve and test the assessment of change in HRQoL over time in these patients.

This study has several strengths. Most patients completed all questionnaires while linear mixed models provide valid estimates of HRQoL in the presence of missing measurements. We investigated both generic and disease-specific HRQoL, rendering a comprehensive picture of HRQoL. We included consecutive patients from two major cardiology referral centres. Our study had a longitudinal design with a baseline measurement prior to the coronary revascularization and three follow-up measurements. Furthermore, the use of two measures of comorbidity burden provides robust results. Finally, comorbidity data were collected by electronic medical records and if unclear were checked by two medical specialists rather than relying on self-report measures of comorbidity.

## Conclusions

Female patients reported poorer HRQoL and their physical health improved more slowly after coronary revascularization than male patients. Higher comorbidity burden was associated with poorer physical health and disease-specific HRQoL in male patients only. Our results provide further insight that female and male patients recover differently after coronary revascularization. These findings also highlight the importance for comorbidity- and gender-specific approaches for evaluating CAD and coronary revascularization procedures.

## Supporting information

S1 Table Identified comorbidity conditions. (DOCX)

S1 Data Minimal data set. (TXT)
