# Correction: Gender differences in quality of life in coronary artery disease patients with comorbidities undergoing coronary revascularization

**DOI:** 10.1371/journal.pone.0307984

**Published:** 2024-07-24

**Authors:** Tom H. Oreel, Pythia T. Nieuwkerk, Iris D. Hartog, Justine E. Netjes, Alexander B. A. Vonk, Jorrit Lemkes, Hanneke W. M. van Laarhoven, Michael Scherer-Rath, Mirjam A. G. Sprangers, José P. S. Henriques

The columns of the parameters beta (β) and standard errors (SE) in Table 2 are incorrect. It should have been chi-square statistics “χ2” and “df” respectively. Please see the correct Table 2 here.

**Table 2:** Wald type II chi-square statistics of the linear mixed models for each HRQOL measure.

Variables	χ ^2^	df	*P* value
***Physical Health (PCS)***			
Time	95.024	1	< .001*
Gender	15.049	1	< .001*
Time × gender	4.843	1	.028*
Comorbidity burden:			
Total comorbidity conditions	16.819	2	< .001*
Charlson comorbidity categories	12.798	2	.002*
Comorbidity burden × gender:			
Total comorbidity conditions	9.448	2	.009*
Charlson comorbidity categories	3.715	2	.156
Comorbidity burden × time:			
Total comorbidity conditions	0.348	2	.840
Charlson comorbidity categories	0.990	2	.610
***Mental health (MCS)***			
Time	44.794	1	< .001*
Gender	1.351	1	.245
Time × gender	0.206	1	.650
Comorbidity burden:			
Total comorbidity conditions	2.143	2	.343
Charlson comorbidity categories	4.787	2	.091
Comorbidity burden × gender:			
Total comorbidity conditions	2.775	2	.250
Charlson comorbidity categories	5.135	2	.077
Comorbidity burden × time:			
Total comorbidity conditions	4.823	2	.090
Charlson comorbidity categories	2.344	2	.310
***Disease-specific HRQoL (SAQ)***			
Time	248.309	1	< .001*
Gender	15.787	1	< .001*
Time × gender	1.564	1	.211
Comorbidity burden:			
Total comorbidity conditions	2.035	2	.362
Charlson comorbidity categories	7.115	2	.029*
Comorbidity burden × gender:			
Total comorbidity conditions	13.761	2	.001*
Charlson comorbidity categories	3.132	2	.209
Comorbidity burden × time			
Total comorbidity conditions	6.877	2	.032*
Charlson comorbidity categories	0.906	2	.636

*P values < 0.05 were considered significant.
