# Supplementary material for: Gender differences in quality of life in coronary artery disease patients with comorbidities undergoing coronary revascularization
Source: PLoS One. 2020 Jun 17;15(6):e0234543. doi: 10.1371/journal.pone.0234543 (PMC7299316; doi:10.1371/journal.pone.0234543)
Supplement: S1 Table — (DOCX) [file pone.0234543.s001.docx]

| Identified comorbidity conditions | Number of patients (N=230) |
| --- | --- |
| Diabetes mellitus | 106 |
| BMI > 30 kg/$\mathbf{m}^{\mathbf{2}}$ | 81 |
| Chronic obstructive pulmonary disease (COPD) | 40 |
| Osteoarthritis (OA) | 25 |
| Hypothyroidism | 23 |
| Cerebral infarction | 18 |
| Gout | 17 |
| Cancer | 16 |
| Dermatitis | 15 |
| Asthma | 13 |
| Obstructive sleep apnea | 12 |
| Arthritis | 10 |
| Kidney failure | 9 |
| Psoriasis | 7 |
| Hearing loss | 7 |
| Glaucoma | 6 |
| Joint disease other | 5 |
| Bronchitis | 4 |
| Neuropathy | 4 |
| Autoimmune disease | 3 |
| Fibromyalgia | 3 |
| Migraine | 3 |
| Tinnitus | 3 |
| Paresis | 3 |
| Chronic lymphocytic leukemia | 2 |
| Chronic pain | 2 |
| Cluster headache | 2 |
| Ankylosing spondylitis | 2 |
| Crohn's disease | 2 |
| Lyme’s disease | 2 |
| Anemia | 1 |
| Barrett's esophagus | 1 |
| Benign prostatic hyperplasia | 1 |
| Blepharospasm | 1 |
| Thoracic outlet syndrome | 1 |
| Chronic suppurative otitis media | 1 |
| Dermatomyositis | 1 |
| Dystonia | 1 |
| Paraplegia | 1 |
| Failed back surgery syndrome | 1 |
| Thymoma with immunodeficiency | 1 |
| HIV | 1 |
| Hemochromatosis | 1 |
| Clubfoot | 1 |
| Myasthenia gravis | 1 |
| Myelodysplastic syndrome | 1 |
| Myositis | 1 |
| Multipele sclerosis | 1 |
| Parkinson | 1 |
| Benign prostatic hyperplasia | 1 |
| Radiculopathy | 1 |
| Rheumatism | 1 |
| Rhinitis | 1 |
| Scoliosis | 1 |
| Stoma | 1 |
| Ménière's disease | 1 |
| Catheter | 1 |
